# Environmental Interventions to Promote Infection Control in Senior-Living Facilities: Cases in the USA and China

**DOI:** 10.1093/geroni/igaa057.1556

**Published:** 2020-12-16

**Authors:** Zhe Wang, Bo Li

**Affiliations:** Henan University, Kaifeng, China

## Abstract

In the evolving situation of COVID-19, an outbreak of the contagious disease in a nursing home near Seattle prompted urgent calls for precautionary tactics in senior-living facilities. Worldwide, the USA and China are the countries with the largest and second largest populations of people aged 80+. More than two million older adults live in senior-living facilities in the USA and the number of beds in senior-living facilities in China has increased to 6.5 million in recent five years. The risk of infections including COVID-19 is tripled in senior-living residents because of age, close living conditions, and underlying health conditions. Infectious diseases account for one third of all deaths in people age 65+ in the USA. COVID-19 has a case fatality rate of 2.3% overall and as high as 15% in patients age 80+ in China. Together with health and services interventions, environmental interventions should be considered to prevent the possible spread of infections in senior-living settings. Based on a literature review and using empirical data of 12 senior-living facility designs collected in both countries, this research analyzed the advantages and disadvantages of existing facility environments in terms of infection control. Environmental interventions to promote infection control are suggested in the context of individual facilities. Proposed interventions are analyzed at two levels: spatial design and design details. Five factors are included: visitors screening, ventilation, isolation rooms, hand washing, and daily temperature checks. From the perspective of infection control, the cross-country similarities and differences of senior-living facility designs are discussed.

